# The Rare Presentation of Spontaneous Pneumothorax in a Pediatric Patient

**DOI:** 10.7759/cureus.41359

**Published:** 2023-07-04

**Authors:** Alison Cullin, Mary-Kate Voit

**Affiliations:** 1 Emergency Medicine, Inspira Medical Center Mullica Hill, Mullica Hill, USA

**Keywords:** asthma, influenza a, subcutaneous emphysema, emergency medicine, spontaneous pneumothorax, pediatric

## Abstract

Children presenting to the Emergency Department (ED) with upper respiratory infection (URI) symptoms of unresolved cough are not uncommon. Differentiation of the child’s symptoms with thorough history and physical and when appropriate, further evaluation with blood work and imaging is the responsibility of the ED physician. In a clinical environment with increasing ED visits due to nonspecific URIs in children, it is also important not to overutilize an emergent workup with unnecessary testing. Our case involves a patient with atypical symptoms and hopes to highlight the importance of keeping a broad differential for all patients upon initial evaluation. Spontaneous pneumothorax is a potentially life-threatening condition. Our five-year-old patient presented with an unresolved cough being treated by an outpatient physician. He had no prior airway disease diagnosis. Severe cough in the absence of prior airway disease is notably less likely, but not an unseen cause of spontaneous pneumothorax. Our patient, however, developed just that. He was officially diagnosed with a right-sided pneumothorax on chest x-ray, underwent supplemental oxygen therapy, and was ultimately transferred to a pediatric hospital for continuation of care. Once there, our patient gradually improved was diagnosed as an asthmatic, and was started on appropriate maintenance medications. It is important to remain vigilant when examining multiple pediatric patients in a shift and to keep in mind that even otherwise healthy pediatric patients are at risk for spontaneous pneumothorax.

Spontaneous pneumothorax is a potentially life-threatening condition. Our five-year-old patient had no prior airway disease diagnosis making spontaneous pneumothorax notably less likely, however, from severe cough our patient developed just that. He was officially diagnosed with right-sided pneumothorax, underwent supplemental oxygen therapy, and was transferred to a pediatric hospital. Once there patient gradually improved, he was diagnosed as an asthmatic and started on appropriate medication to keep his breathing stable. It is essential to keep in mind that even otherwise pediatric patients are at risk for spontaneous pneumothoraxes and we as emergency physicians must keep this in mind during our evaluation.

## Introduction

Pneumothorax is defined as the pathological collection of free air in the potential space between the visceral and pleural lining known as the pleural space [1]. The potential development of a life-threatening tension pneumothorax makes this a diagnosis that requires prompt recognition and intervention. Categories of pneumothorax include spontaneous, iatrogenic, and traumatic. Spontaneous pneumothorax is further broken down into primary (occurring secondary to blebs/bullae without other lung pathology) and secondary (occurring in relation to underlying acute and/or chronic lung diseases) [1-3]. A spontaneous pneumothorax is a relatively rare condition in children with the nadir of cases occurring in the neonatal or late adolescent period [2,3]. Commonly known predisposing conditions for secondary spontaneous pneumothorax include ranging from asthma and foreign body aspiration to cystic fibrosis and chronic infectious etiologies. In such conditions, it is thought that chronic airway inflammation in smaller airways leads to obstructions that create increased pressures leading to air escape into the pleural space, and are then more susceptible to pneumothorax development [3].  

The incidence of primary spontaneous pneumothorax in pediatric patient populations is estimated at 3.4/100,000 as opposed to the general adult population with 6-18/100,000 each year with a predominance towards males after eight years of age [3,4]. Preadolescent primary and secondary spontaneous pneumothorax incidence are approximately equivalent. The peak age of incidence is the neonatal period secondary to developmental abnormalities, 16-24 years of age, and then again in ages greater than 55 because of acquired lung pathology over the patient’s lifetime [2]. Upon presentation, a large pneumothorax is likely to include symptoms of tachypnea, sharp/stabbing chest pain on the side of the pneumothorax, and dyspnea. Smaller pneumothoraces may be asymptomatic and can be an incidental finding. The extent of the pneumothorax, clinical presentation, and underlying cause determines treatment. In smaller pneumothoraces, supplemental oxygen is often appropriate. However, larger cases may require a chest tube and more invasive surgical intervention including video-assisted thoracoscopic surgery (VATS) along with treatment of the underlying cause [3]. Fortunately, our patient required non-invasive management, and clinical improvement was achieved without surgical intervention. This favorable outcome is in part due to prompt diagnosis and treatment prior to clinical deterioration. 

## Case presentation

**Figure 1:**
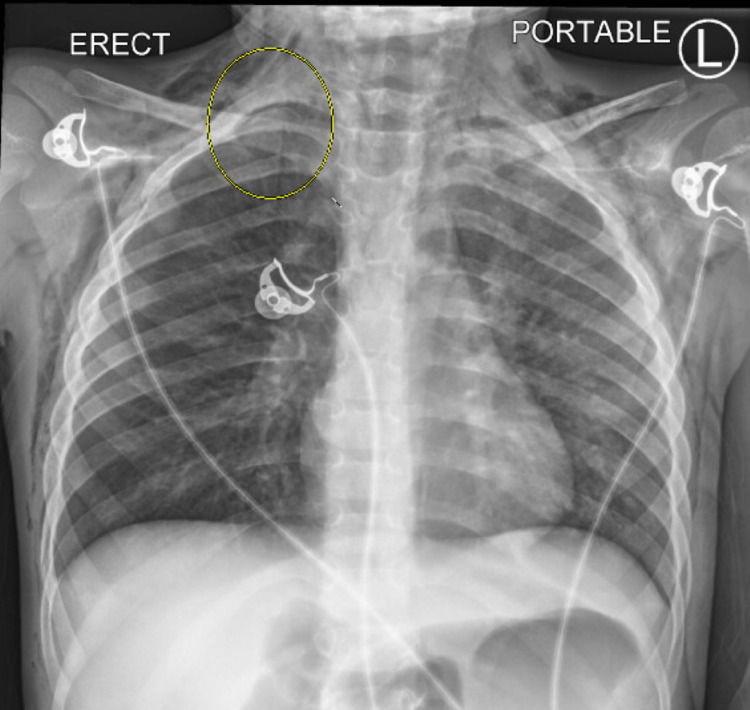
Radiology read: Lungs are clear. Tiny pneumothorax right lung apex. Mild surface emphysema of the both chest wall and base of the neck in both sides. Heart is normal in size.

A five-year-old male with no reported past medical history presented to the ED with a chief complaint of increasing shortness of breath. The patient had presented to urgent care (UC) one day prior with a three-day history of cough and shortness of breath. The patient’s father reported a max fever of 39.4°C and oxygen(O2) saturation of 93% on room air (RA). Our patient received viral testing and a chest x-ray, was administered steroids and two albuterol treatments via a nebulizer, and was diagnosed with influenza A and pneumonia while at urgent care. He was discharged to home on Amoxicillin and continued home nebulizers as he had a machine from a prior respiratory infection. As per his father, by the morning our patient had worsening shortness of breath, increased work of breathing, was not acting like himself, and was speaking nonsense. The home nebulizer was not helping, and the patient had difficulty tolerating the mask on his face. Upon arrival at the emergency department (ED), he was noted to be tachypneic with a respiratory rate of 48 breaths per minute and an O2 saturation of 88% on RA. He was immediately placed on a 3L nasal cannula (NC) with improvement in O2 saturations to the low-mid 90s and continued tachypnea. On physical examination, our patient was in moderate respiratory distress with subcostal retractions and accessory muscle use. Lung sounds were diminished on the right compared to the left and crackles were noted in the right lower lung field. Chest wall exam yielded mild palpable crepitus bilaterally. Further evaluation with labs and repeat chest x-ray were ordered. Lab evaluation was grossly unremarkable, aside from positive Influenza A. Chest x-ray was obtained and demonstrated a right apical pneumothorax with subcutaneous emphysema (Figure 1). While in the ED, our patient transitioned to a high-flow nasal cannula (HFNC) with continued improvement in O2 saturation and work of breathing. There was no indication for chest tube placement given on the small size of the pneumothorax without evidence of tension physiology along with his improving clinical status. Our patient was emergently transferred from our ED to a local pediatric intensive care unit for continued close monitoring and further treatment. 

Upon review of the patient’s hospital course, he was admitted to the intensive care unit on 12L HFNC with a 40% fraction of inspired oxygen (FiO2) with a simple mask for improved patient compliance. Pulmonology was consulted and recommended starting methylprednisolone 1mg/kg/dose intravenous every six hours with continuous albuterol nebulizers. The patient remained nothing by mouth (NPO) and had dextrose 5% in normal saline with added potassium chloride started at a maintenance rate. The patient was also started on fluticasone 44mcg two puffs twice a day, Oseltamivir 45mg by mouth every 12 hours for five days, and ampicillin-sulbactam 100mg ampicillin/kg/day divided for every six hours. Repeat chest x-ray the next morning demonstrated remaining pneumothorax. However, a repeat chest x-ray that afternoon showed a resolution of pneumothorax, but, revealed pneumomediastinum and worsening subcutaneous air. He was transitioned to oral amoxicillin-clavulanate 45mg/kg/day divided every 12 hours and supplemental oxygen was gradually decreased. Our patient was weaned to room air within the next 24 hours and pneumomediastinum remained stable. He was discharged to home on fluticasone 44mcg two times per day, Albuterol inhaler one/two puffs every four hours, oral prednisolone 1mg/kg/day for five days, oral amoxicillin-clavulanate 45mg/kg/day and oral Oseltamivir 45mg twice per day for three more days. The patient was instructed to follow up at the pediatric pulmonology clinic in four weeks for a repeat chest x-ray. Based on the presentation, pulmonology services at the pediatric facility related this incidence to undiagnosed asthma, however, no spirometry testing was completed in the acute setting. Official diagnoses at the time of discharge included acute hypoxic respiratory failure secondary to influenza A, status asthmaticus secondary to influenza A with superimposed bacterial pneumonia, pneumothorax, and pneumomediastinum. 

## Discussion

Pneumothorax can be a life-threatening condition especially if missed and/or progresses to a tension pneumothorax. Emergency medicine providers must remain highly suspicious especially with atypical presentations and in less common age groups. There are multiple types of pneumothoraces including iatrogenic, traumatic, and spontaneous which are then broken down into primary and secondary spontaneous pneumothorax [3]. Spontaneous pneumothorax is rare in the age group between neonatal and adolescence, as developmental causes are likely to have caused a pneumothorax shortly after birth and adolescence can lead to rapid stretching during growth spurts [4]. Upon presentation, while some may be asymptomatic, commonly patients have dyspnea, tachypnea, tachycardia, sharp chest pain, and hypoxia. As intrathoracic pressures accumulate, and tension pneumothorax develops patients will develop further evidence of not only respiratory but also cardiovascular compromise secondary to the decreased blood return to the heart from the decreased pressure gradient [4]. Vital signs will thus begin to demonstrate hypotension and because of these changes, this is an emergent diagnosis. 

Immediate intervention is required and may range anywhere from supplemental oxygen via nasal cannula to large-bore chest tube. Determination of intervention for spontaneous pneumothorax is dependent upon primary versus secondary, size, vital signs, and overall patient presentation. In secondary cases not only does the pneumothorax need treatment but also the underlying lung pathology [2]. For example, in a patient presenting with asthma and pneumothorax the supplemental oxygen given to assist with opening and keeping open collapsed lung tissue will not be able to keep up until the exacerbation is managed. Another key point in management is to avoid high-pressure circuits such as continuous positive airway pressure (CPAP) as this will pump further air into the pneumothorax thus collapsing the lung further and worsening the patient’s stability. While CPAP may be necessary, a chest tube must be placed prior to initiation to prevent patient decline [3]. 

In our patient’s situation, not only is he five years old, a rare age group, but he also did not have a prior diagnosis of asthma according to the history provided by his father. He would have been considered a primary spontaneous pneumothorax suspected to be related to intrathoracic pressure changes during extensive coughing fits. While the patient was hypoxic with increased work of breathing, CPAP was held until a chest x-ray could be obtained. Thankfully, a portable chest x-ray was obtained quickly, and no CPAP was initiated based on the image as this would have worsened not only the pneumothorax but also the subcutaneous emphysema and pneumomediastinum. The priority became supplemental oxygen without continuous pressure. Breathing treatments were provided for any inflammation related to influenza and the consideration of underlying undiagnosed asthma. This allowed the patient to have an improvement in hypoxia and work of breathing. Based on his final diagnosis at the time of discharge from the pediatric hospital patient’s category would be secondary spontaneous pneumothorax as he was officially diagnosed with asthma. These types of presentations are uncommon, especially in this age group, leading to the importance of continued vigilance, being informed of treatment pathway progression, and continued research into the management of an atypical pediatric patient presenting with a pneumothorax. 

## Conclusions

In conclusion, spontaneous pneumothoraces in five-year-olds is a rare condition, however, can be life-threatening. This is something that must be considered regardless of prior medical history or trauma history. Based on the extent of the pneumothorax and the patient’s presentation will dictate the required intervention. This can be anything from supplemental oxygen and observation all the way to chest tube insertion(s) and possible surgical correction. Thus, spontaneous pneumothorax is a condition that emergency physicians must always have a high suspicion for.  
